# Long‐term mid‐onset dietary restriction rejuvenates hematopoietic stem cells and improves regeneration capacity of total bone marrow from aged mice

**DOI:** 10.1111/acel.13241

**Published:** 2020-09-15

**Authors:** Si Tao, Yiting Wang, Jianying Wu, Ting Zeng, Hui Cui, Zhendong Tao, Lang Lei, Li Yu, Anwen Liu, Hua Wang, Liu Zhang, Duozhuang Tang

**Affiliations:** ^1^ Department of Oncology Jiangxi Key Laboratory of Clinical and Translational Cancer Research The Second Affiliated Hospital of Nanchang University Nanchang China; ^2^ Department of Oncology The Second Affiliated Hospital of Nanchang University Nanchang China; ^3^ Department of Hematology The Second Affiliated Hospital of Nanchang University Nanchang China; ^4^ Department of Medical Laboratory Medicine Jiangxi Province Hospital of Integrated Chinese & Western Medicine Nanchang China; ^5^ Department of Pathology The Second Affiliated Hospital of Nanchang University Nanchang China; ^6^ Intensive Care Unit Peking University People's Hospital Beijing China

**Keywords:** aging, bone marrow transplantation, dietary restriction, hematopoietic stem cells

## Abstract

Currently, the world's aging population is expanding rapidly, leading to a rise in aged hematopoietic cell transplantation (HCT) recipients and aged donors. However, the age of donors is negatively related to the prognosis after transplantation due to functional decline in hematopoietic stem cells (HSCs) during aging. Previously, we showed that an early‐onset dietary restriction (DR) significantly retards early aging of HSCs. However, the effects of a mid‐onset DR on HSCs remain unknown. In the current study, we performed 30% DR in 15‐ to 18‐month‐old mice (equivalent to 50–60 human years) for short‐term (4 months) and long‐term (9 months). We show that DR reduces and rectifies the imbalance of the HSC pool in aged mice. Short‐term DR improves hematopoietic reconstitution in purified HSC transplantations, but not in bone marrow transplantations. Intriguingly, long‐term mid‐onset DR improves the hematopoietic regeneration of aging HSCs with a particular enhancement of lymphoid outputs even in total bone marrow transplantation settings. Mechanistically, long‐term DR rejuvenates the aberrantly regulated mitochondrial pathways in aging HSCs and is accompanied by increased quiescence and reduced DNA damage signaling in HSCs. Short‐term DR showed a similar trend of rescuing these aging hallmarks but to a much lesser extent. Together, the current study suggests that mid‐onset DR ameliorates the function of aging HSCs and long‐term DR even improved hematopoietic reconstitution in bone marrow transplantation, which could potentially have considerable implications in HCT of humans when only old donors are available.

## Graphical abstract

The study shows that mid‐onset DR rectifies the imbalance of HSC pool in aged mice and leads to suppression of B lymphopoiesis in undisturbed conditions. Short‐term mid‐onset DR improves the hematopoietic regeneration of aging HSCs and long‐term DR even improved repopulation capacity of total bone marrow cells. Mechanistically, DR rejuvenates the aberrantly regulated mitochondrial pathways accompanied by increased quiescence and reduced DNA damage signaling.

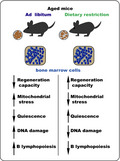

## 1 INTRODUCTION

Hematopoietic cell transplantation (HCT) is a key treatment for curing many hematopoietic malignancies. The world's aging population has been expanded remarkably, resulting in a rise in aged HCT recipients and donors (Visram et al., 2018). However, for every 10‐year increment over 60 years in donor age, there is a 5.5% increase in the hazard ratio for overall mortality after HCT (Kollman et al., 2016). Aged HSCs exhibit reconstitution decline, particularly in lymphoid regeneration (Lau, Kennedy, Kirkland, & Tullius, 2019). Therefore, exploring ways to improve the repopulating capacity of HSCs from old donors could have considerable implications in HCT (Arai et al., 2016; Gonzalez‐Vicent et al., 2017).

Previously, we have shown that an early‐onset dietary restriction (DR) improved HSC repopulating capacity in early aging of C57BL/6 J mice (Tang et al., 2016). However, the effects of a mid‐onset DR on sorted HSCs and unpurified hematopoietic cells which are dominantly used in clinical HCT have not been reported so far.

In this study, we analyzed the effects of a 30% DR regimen applied for short‐term (4 months) and long‐term (9 months) in C57BL/6 J mice starting at 15–18 months of age (the equivalent change in humans would roughly be 50–60 years (Dutta & Sengupta, 2016)) on the function of HSCs and bone marrow cells in transplantations as well as in undisturbed conditions.

### 1.1 Short‐term DR rejuvenates aged HSCs but only long‐term DR benefits in bone marrow transplantation

**Figure 1:**
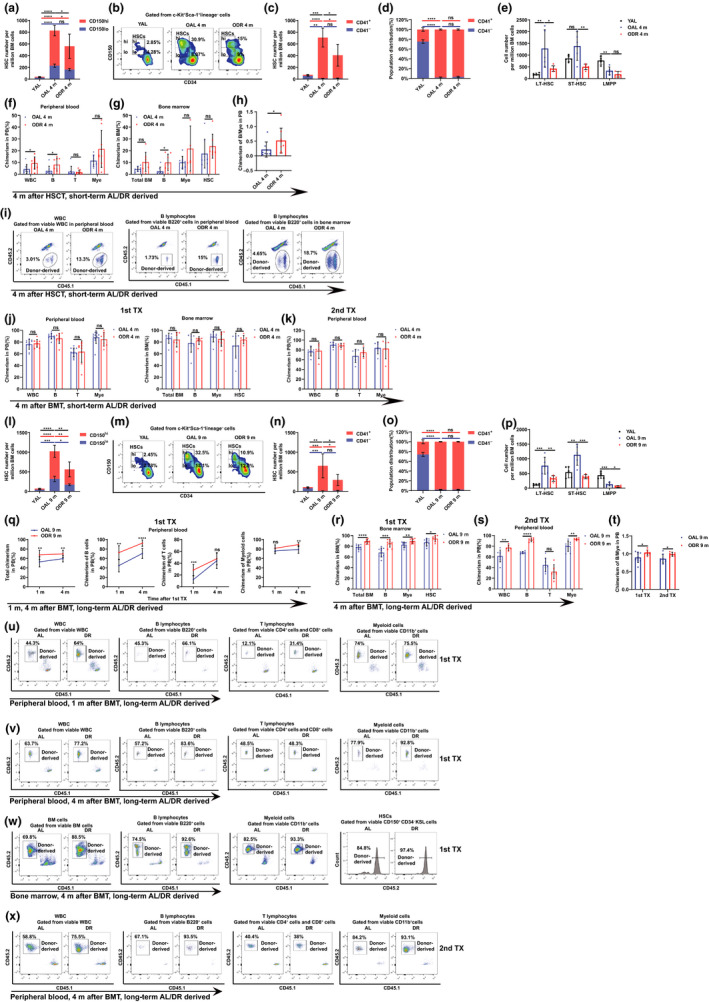
Short‐term DR rejuvenates aged HSCs but only long‐term DR benefits in bone marrow transplantation. Mice were fed with DR or AL diet for 4 months (OAL 4 m/ODR 4 m, a–k) or 9 months (OAL 9 m/ODR 9 m, l–x). (a–e and l–p) Frequencies of indicated populations in 4 months (a–e) or 9 months (l–p) treated mice determined by FACS (n = 4–7). (f–k and q–x) Competitive transplantation analysis. Donor‐derived chimerisms and representative FACS plots in peripheral blood and bone marrow in recipients after HSC transplantation (f,g,i, n = 7–8 recipient mice per group) or bone marrow transplantation (j,k, short‐term treatment, n = 5–11 recipient mice per group; (q–s and u–x) long‐term treatment, n = 6–11 recipient mice per group). (h and t) The ratio of donor‐derived chimerism in B cells versus myeloid cells in short‐term HSCT (h) and long‐term BMT (t). Results were displayed as mean ± SD. ns, not significant; *p < 0.05; **p < 0.01;***p < 0.001;****p < 0.0001. (f–h,j,k,q–t) Unpaired two‐tailed Student's t test; (a,c–e,l,n–p) one‐way ANOVA. Data show results from 2 independent experiments. YAL, young control

Short‐term DR significantly reduced the size of HSC pool in the old mice, in particular, the myeloid‐biased HSCs (CD150^high^HSCs and CD41^+^HSCs), whereas the lymphoid‐biased HSCs (CD150^low^HSCs and CD41^−^HSCs) was not altered (Figure 1a–d). DR also significantly rescued the aging‐associated change in frequencies of LT‐HSCs but failed to rescue the diminishment of LMPPs (Figure 1e).

To study the impact of DR on HSC function, purified HSCs from the above treated mice were transplanted along with competitor cells. Interestingly, DR‐derived HSCs led to higher chimerisms in whole white blood cells (WBC) and B lymphocytes, and the ratio of chimerism in B cells/chimerism in myeloid cells, with similar chimerisms in myeloid cells (Figure 1f–i). However, clinical HCT dominantly use unpurified hematopoietic cells as graft sources. Although short‐term DR improves regeneration of HSCs *per se*, it also reduces the frequency of HSCs which may have negative effect on HCT (Figure 1a). Of note, the short‐term DR showed neutral effects in bone marrow transplantations (BMT), indicating that the functional improvement achieved by short‐term DR could not compensate reduced frequency of HSCs (Figure 1j,k). We further performed another competitive BMT using bigger number of bone marrow cells from DR donors to reach same amount of HSCs to be transplanted as from AL donors. In this experiment, DR donor‐derived bone marrow generated higher chimerisms in lymphoid and myeloid cells in competitive serial transplantations (Figure S1).

We further studied the effect of a longer‐term (9 months) DR on aged mice. Comparing to short‐term DR, long‐term DR showed similar effects on HSCs and progenitors and significant reduction of CD150^low^HSCs (Figure 1l–p). Intriguingly, long‐term DR increased the chimerisms in both B cells and myeloid cells in competitive BMT (Figure 1q–x). Furthermore, DR donor generated higher ratio of chimerism in B cells/chimerism in myeloid cells (Figure 1t). Notably, the beneficial effect on lymphocytes starts as early as 1 month after BMT, which is essential in early anti‐infection activities post‐transplantation (Figure 1q,u).

### 1.2 DR ameliorates development of aging hallmarks in HSCs

**Figure 2:**
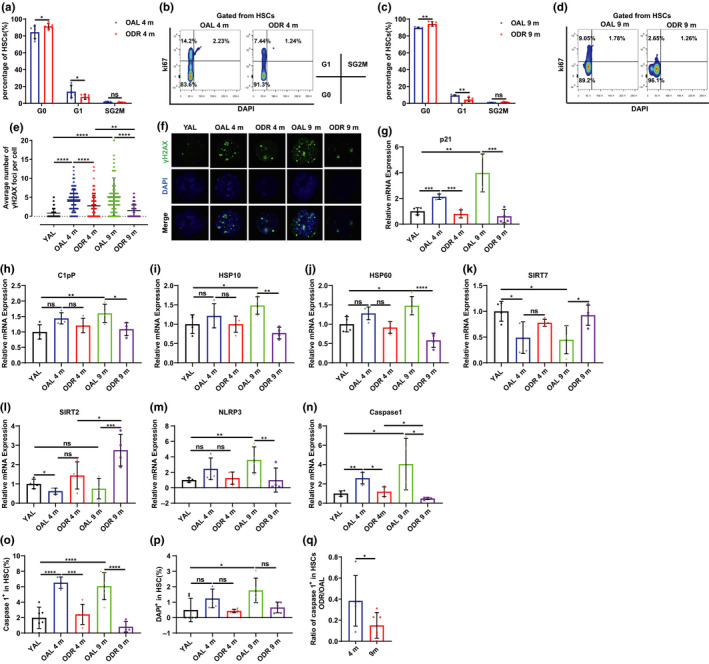
DR ameliorates development of aging hallmarks in HSCs. Mice were fed with DR or AL diet for 4 or 9 months. (a–d) Quantification of HSCs in indicated cell cycle phases (n = 5–7) and representative FACS plots. (e) Statistical analysis and (f) representative images of γH2AX in HSCs by immunostaining. (g–n) qPCR analysis of relative mRNA expression of indicated genes in HSCs. (o,p) Frequencies of caspase 1^+^ and DAPI^+^ cells in HSCs (n = 5–6). (q) The ratio of frequencies of caspase 1^+^ cells in HSCs in ODR versus OAL. Results were displayed as mean ± SD. ns, not significant; *p < 0.05; **p < 0.01;***p < 0.001; ****p < 0.0001. (a,c,q) Unpaired two‐tailed Student's t test; (e,o,p) one‐way ANOVA; (g–n) two‐way ANOVA. Data show results from 2 independent experiments

Quiescence is an essential mechanism to protect HSCs against proliferation‐induced damage and deteriorations in their functionality (Hu & Wang, 2019; Walter et al., 2015; Wilson et al., 2008). Exit from quiescent stage leads to accumulation of DNA damage and functional decline in HSCs (Walter et al., 2015). HSCs from DR mice showed a significant increase in the percentage at G0 phase and a decrease in the G1 phase and, correlatively, reduced formation of γ‐H2AX foci and expression of p21 (Figure 2a–g). HSC quiescence is mediated by mitochondrial unfolded protein response (UPR^mt^) which is increased upon elevated mitochondrial protein folding stress (PFS^mt^) during aging (Mohrin et al., 2015; Mohrin, Widjaja, Liu, Luo, & Chen, 2018). Furthermore, with age, reduced Sirt2 expression and increased mitochondrial stress lead to aberrant activation of NLRP3 and caspase 1, which impairs HSC regenerative capacity (Luo et al., 2019). Notably, long‐term DR remarkably rejuvenated expression of UPR^mt^ genes (C1pP, HSP10 and HSP60), the UPR^mt^ upstream suppressor SIRT7, and the SIRT2‐NLRP3‐caspase 1 axis in aging HSCs to levels approaching that of young HSCs; short‐term DR also showed a similar trend of rescuing these aging hallmarks but to a much lesser extent (Figure 2h–n). In line with that, aging‐associated increase of caspase 1‐induced‐pyroptosis was decreased under DR (Figure 2o,q). The rate of DAPI^+^ cells was not changed by DR (Figure 2p).

### 1.3 DR suppresses B lymphopoiesis in donor mice in undisturbed conditions

We further analyzed the effect of DR on downstream lineages in donor mice in undisturbed conditions. DR showed minor effects on bone marrow cells, peripheral blood WBCs, and myeloid lineages but the B lymphoid lineage and proliferation of lymphoid progenitors were severely suppressed, similar to our previous findings in an early‐onset DR (Figure S2a–x) (Tang et al., 2016). The ratio of lymphoid versus myeloid cells (L/M) in peripheral blood was decreased during aging and was not rescued by DR (Figure S2y).

The current study demonstrates for the first time that DR acts on aging HSCs which already harbor accumulated molecular damage. Both short‐term and long‐term mid‐onset DR significantly rectifies the imbalance in the HSC pool but only long‐term DR benefits in bone marrow transplantations, indicating long‐term DR exerts stronger effects on HSCs. Particularly, DR in donor mice retards aging‐associated decline in lymphopoiesis in HCT. The findings could potentially have considerable implications in HCT of humans when only old donors are available, where deficiency in repopulating activities is a major concern (Arai et al., 2016; Gonzalez‐Vicent et al., 2017).

Mid‐onset DR showed a severe suppression in B lymphopoiesis in donor mice in undisturbed conditions. We hypothesis that the discrepancy in B lineage outputs under steady‐state hematopoiesis compared to transplantation condition is because the systemic factors (IL‐7 and IL‐6) were down‐regulated in donors receiving DR, while they were normally expressed in recipients which were fed ad libitum, as what we have shown previously (Tang et al., 2016).

Mechanistically, we show that long‐term DR rejuvenates the aberrantly regulated mitochondrial pathways in aging HSCs and is accompanied by increased quiescence and reduced DNA damage signaling in HSCs. Short‐term DR showed a similar trend of rescuing these aging hallmarks but to a much lesser extent.

## 2 EXPERIMENTAL PROCEDURES

Experimental procedures are available online as supporting information.

### 2.1 Mice and dietary restriction

C57BL/6 J mice were obtained from Hunan SJA Laboratory Animal Co. Ltd. and maintained in the animal facilities of Nanchang Royo Biotech under pathogen‐free conditions on a 12‐h light/12‐h dark cycle at 23°C–25°C. DR was performed as previously described (Tang et al., 2016). Briefly, mice were housed individually and fed with a timed daily supply of food—70% of the normal food amount that the body weight—and gender‐matched ad libitum (AL) mice consumed. For the AL group, mice were fed with an unlimited amount of food. All mouse experiments were approved by the Animal Experimental Ethical Inspection of Nanchang Royo Biotech Co. Ltd (RYEI20170513‐1). Three‐month‐old mice were used as young control, and DR was initiated from 15–18 months of age (the equivalent change in humans would roughly be 50–60 years (Dutta & Sengupta, 2016)).

### 2.2 Flow cytometry and HSC sorting

The following combinations were used for indicated populations: HSCs (CD150^+^CD34^−^c‐Kit^+^Sca‐1^+^lineage^−^ cells), myeloid‐biased HSCs (CD150^high^ CD34^−^c‐Kit^+^Sca‐1^+^lineage^−^ cells), lymphoid‐biased HSCs (CD150^low^ CD34^−^c‐Kit^+^Sca‐1^+^lineage^−^ cells), CD41^+^ HSCs (CD41^+^CD150^+^CD34^−^c‐Kit^+^Sca‐1^+^lineage^−^ cells), CD41^−^ HSCs (CD41^−^CD150^+^CD34^−^c‐Kit^+^Sca‐1^+^lineage^−^ cells), LT‐HSCs (Flt3^−^CD34^−^c‐Kit^+^Sca‐1^+^lineage^−^ cells), ST‐HSCs (Flt3^−^CD34^+^c‐Kit^+^Sca‐1^+^lineage^−^ cells), LMPPs (Flt3^high^CD34^+^c‐Kit^+^Sca‐1^+^lineage^−^ cells),common myeloid progenitors (CMPs; CD16/32^−^CD34^+^c‐Kit^+^Sca‐1^−^lineage^−^ cells), megakaryocyte/erythrocyte progenitors (MEPs; CD16/32^−^CD34^−^c‐Kit^+^Sca‐1^−^lineage^−^ cells), granulocyte/macrophage progenitors (GMPs; CD16/32^+^CD34^+^c‐Kit^+^Sca‐1^−^lineage^−^ cells), common lymphoid progenitors (CLPs; IL‐7Rα^+^Flt3^+^c‐Kit^mid/low^Sca‐1^mid/low^lineage^−^ cells), and progenitor B cells (pro‐B cells, B220^+^CD24^+^AA4.1^+^TER‐119^−^Gr‐1^−^CD11b^−^CD3^−^ cells).

BM cells were obtained by crushing all hind limbs and pelvis with sterile PBS and filtered with 40‐μm cell strainer. Afterward, the BM cells were resuspended in red cell lysis buffer and incubated at room temperature for 5 min to lyse the red cells. Then, BM cells were washed with PBS, counted by the cell counter, and were incubated with different combinations of antibodies for flow cytometry analysis. The following antibodies were used: a lineage cocktail for HSPCs, CMPs, MEPs, GMPs, and CLPs (biotinylated anti‐TER‐119, ‐Gr‐1, ‐B220, ‐CD11b, ‐CD3, ‐CD4, and ‐CD8 antibodies; BioLegend), a lineage cocktail for pro‐B cells (biotinylated anti‐Gr‐1, ‐CD11b, ‐TER‐119, and ‐CD3 antibodies), Alexa Fluor 700‐conjugated anti‐CD34 (eBioscience), FITC‐conjugated anti‐CD34 (BD), APC‐conjugated anti‐CD150 (BioLegend), PerCP‐Cy5.5‐conjugated anti‐CD150 (BioLegend), BV605‐conjugated anti‐CD150 (BioLegend), PE‐Cy7‐conjugated anti‐CD41(BioLegend), PE‐Cy7‐conjugated anti‐c‐Kit (eBioscience), APC‐conjugated anti‐c‐Kit (BioLegend), Pacific blue‐conjugated anti‐Sca‐1 (BioLegend), PE‐conjugated anti‐Sca‐1 (BioLegend), PE‐Cy7‐conjugated anti‐Sca‐1 (BD), FITC‐conjugated anti‐CD16/32 (BioLegend), PE‐Cy7‐conjugated anti‐CD16/32 (BioLegend), PerCP‐Cy5.5‐conjugated anti‐IL‐7Rα (eBioscience), PE‐conjugated anti‐Flt3 (BioLegend), APC‐Cy7‐conjugated anti‐CD19 (BioLegend), PerCP‐Cy5.5‐conjugated anti‐CD19 (BioLegend), Pacific blue‐conjugated anti‐B220 (BioLegend), PE‐Cy7‐conjugated anti‐B220 (BioLegend), PE‐conjugated anti‐CD43 (BD), APC‐conjugated anti‐AA4.1 (BioLegend), FITC‐conjugated anti‐CD24 (BioLegend), APC‐conjugated anti‐TER‐119 (BioLegend), PE‐conjugated anti‐CD71 (BioLegend), PE‐conjugated anti‐B220 (BioLegend), APC‐Cy7‐conjugated anti‐CD11b (BioLegend), FITC‐conjugated anti‐Gr‐1 (eBioscience), APC‐conjugated anti‐CD4 (BioLegend), and PerCP‐Cy5.5‐conjugated anti‐CD8 (BioLegend) antibodies, streptavidin‐APC‐Cy7 (BioLegend), and streptavidin‐Alexa Fluor 700. After staining, cells were analyzed on a flow cytometer (FACS Canto II; BD). For cell cycle analysis, a lineage cocktail as described in the previous paragraph and Alexa Fluor 700‐conjugated anti‐CD34, FITC‐conjugated anti‐CD34 (BD), PE‐conjugated anti‐CD150 (BioLegend), PerCP‐Cy5.5‐conjugated anti‐CD150 (BioLegend), APC‐conjugated anti‐c‐Kit (BioLegend), PE‐Cy7‐conjugated anti‐Sca‐1 (BioLegend), and PerCP‐Cy5.5‐conjugated anti‐CD48 antibodies and streptavidin‐APC‐Cy7 were used. After staining, cells were fixed and permeabilized with the Cytofix/Cytoperm Fixation/Permeabilization Solution kit (BD) according to the manufacturer's instructions. Afterward, cells were incubated with PE‐conjugated anti‐Ki67 antibody (BD) for 1 h on ice and incubated with DAPI/PBS medium to stain for DNA content. For active caspase 1 and DAPI staining, after stained for HSC markers using a lineage cocktail as described in the previous section, 10 µl of diluted FLICA reagents (1:5 dilution of stock in DMSO) was added to 290 µl of bone marrow cells. After incubation for 1 h at 37°C, samples were washed three times with 1 ml 1X apoptosis buffer before flow cytometry analysis according to the manufacturer's instructions and incubated with DAPI/PBS medium.

For HSC sorting, BM cells were pre‐enriched for c‐Kit^+^ cells as described previously (Tang et al., 2016). Briefly, BM cells were incubated with APC‐conjugated anti‐c‐Kit antibody, and c‐Kit^+^ cells were enriched using anti‐APC magnetic beads and LS columns (Miltenyi Biotec). The positively selected cells were then stained for HSC markers using a lineage cocktail as described in the previous section and FITC‐conjugated anti‐CD34 (BD), PerCP‐Cy5.5‐conjugated anti‐CD150 (BioLegend), APC‐conjugated anti‐c‐Kit, and PE‐conjugated anti‐Sca‐1 (BioLegend) antibodies and streptavidin‐APC‐Cy7. After staining, cells were sorted on a cell sorter (FACS Aria III; BD).

All FACS gating strategies are shown in Figure S3.

### 2.3 Transplantation

Bone marrow cells from 5 donor mice from each treatment group were pooled and prepared for the following experiments. For HSC transplantation, 400 FACS‐purified HSCs from Ly5.1 donor mice were mixed with 1 million BM cells (competitor cells) from Ly5.1/5.2 mice and injected via the tail vein into lethally irradiated Ly5.2 recipient mice. For bone marrow transplantation, two different ratios of donor versus competitor cells were used: (1) same amount of bone marrow cells from AL and DR donor mice: 1 million BM cells from Ly5.2 donor mice were mixed with 1 million BM cells (competitor cells) from Ly5.1 mice and injected via the tail vein into lethally irradiated Ly5.1 recipient mice and (2) greater amount of bone marrow cells from DR donor mice compared to AL donor mice to reach same amount of HSCs to be transplanted: 1 million BM cells from Ly5.2 AL mice and 1.7 million BM cells from Ly5.2 DR mice were mixed with 1 million BM cells (competitor cells) from Ly5.1 mice, respectively, and injected via the tail vein into lethally irradiated Ly5.1 recipient mice.

### 2.4 Immunostaining

Cells were sorted and stained as previously described (Ema et al., 2006). Briefly, cells were FACS‐purified and dropped on poly‐L‐lysine‐coated glass slides (Shanghai JingAn Biological) and fixed with 4% paraformaldehyde for 10 min at room temperature (RT), followed by permeabilization in 0.25% Triton/PBS for 10 min at RT and a blocking with 1% BSA/PBS for 1 h at RT. Then, cells were incubated with primary antibody anti‐phospho‐Histone H2AX (Ser139) Antibody (Merck) at 1:500 dilution overnight at 4°C followed by an incubation with secondary antibody anti‐mouse Alexa Fluor488 (Invitrogen) for 1 h at RT. To visualize the nuclei, the cells were counterstained by DAPI. Images were acquired on a Leica SP5 fluorescent microscope and processed by LAS‐AF‐Lite_2.6.0. One hundred and fifty HSCs from 3 samples per group were scored blindly, and foci were counted manually according to previously published protocols (Gutierrez‐Martinez et al., 2018).

### 2.5 RNA Isolation, cDNA Synthesis, and Quantitative Real‐Time PCR

Total RNA was isolated from freshly sorted cells by using RNAsimple Total RNA Kit (TianGen Biotech). TransScript‐Uni One‐Step gDNA Removal and cDNA Synthesis SuperMix (TransGen Biotech) was used for reverse transcriptions. qRT‐PCR was performed with an ABI 7900 Real‐Time PCR System (Applied Biosystems) and TransStart Tip Green qPCR SuperMix (TransGen Biotech). Relative expression of genes was normalized to β‐actin in each sample and was calculated using the ΔCt method. Primer sets are listed in Table S1.

### 2.6 Peripheral blood cell counting

Peripheral blood was collected from the orbital venous plexus containing 5 μl EDTA (0.5 M). PB cell count was performed on automated hematology analyzer (Sysmex, XS‐500i) according to the manufacturer's instructions.

### 2.7 Statistics

GraphPad Prism 7.0 software was used for statistical analysis. The unpaired two‐tailed Student's t test was used for two‐group datasets, and one‐way ANOVA or two‐way ANOVA was used for multi‐group datasets to calculate *p*‐values. All results were displayed as mean ±SD. ns, not significant; *p < 0.05; **p < 0.01; ***p < 0.001; ****p < 0.0001.

## CONFLICTS OF INTEREST

The authors declare no conflicts of interest.

## AUTHORS CONTRIBUTIONS

S.T. and Y.W. performed and analyzed majority of all experiments. T.Z., J. W., and H. C. treated mice and participated in most of the experiments. Z.T. did peripheral blood cell counting. L.L. helped with immunostaining and photographing. A.L. and H.W. helped with mouse irradiation and transplantation. L.Z. and L.Y. helped with experimental design. S.T. and D.T. conceived and designed the experiments. The manuscript was written by S.T. and D.T. and commented on by all other authors.

## Data Availability

Data sharing is not applicable to this article as no new data were created or analyzed in this study.
