# Supplementary figures and images for: Long‐term mid‐onset dietary restriction rejuvenates hematopoietic stem cells and improves regeneration capacity of total bone marrow from aged mice
Source: Aging Cell. 2020 Sep 15;19(10):e13241. doi: 10.1111/acel.13241 (PMC7576247; doi:10.1111/acel.13241)

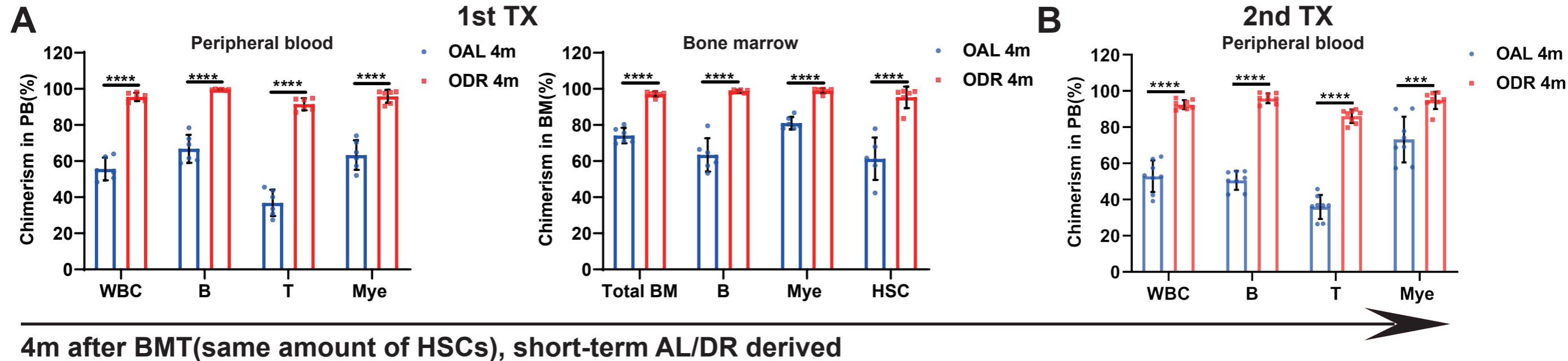

Tao and Wang et al\_Figure S1

Supplement: Supplementary file 1 [file ACEL-19-e13241-s001.pdf]

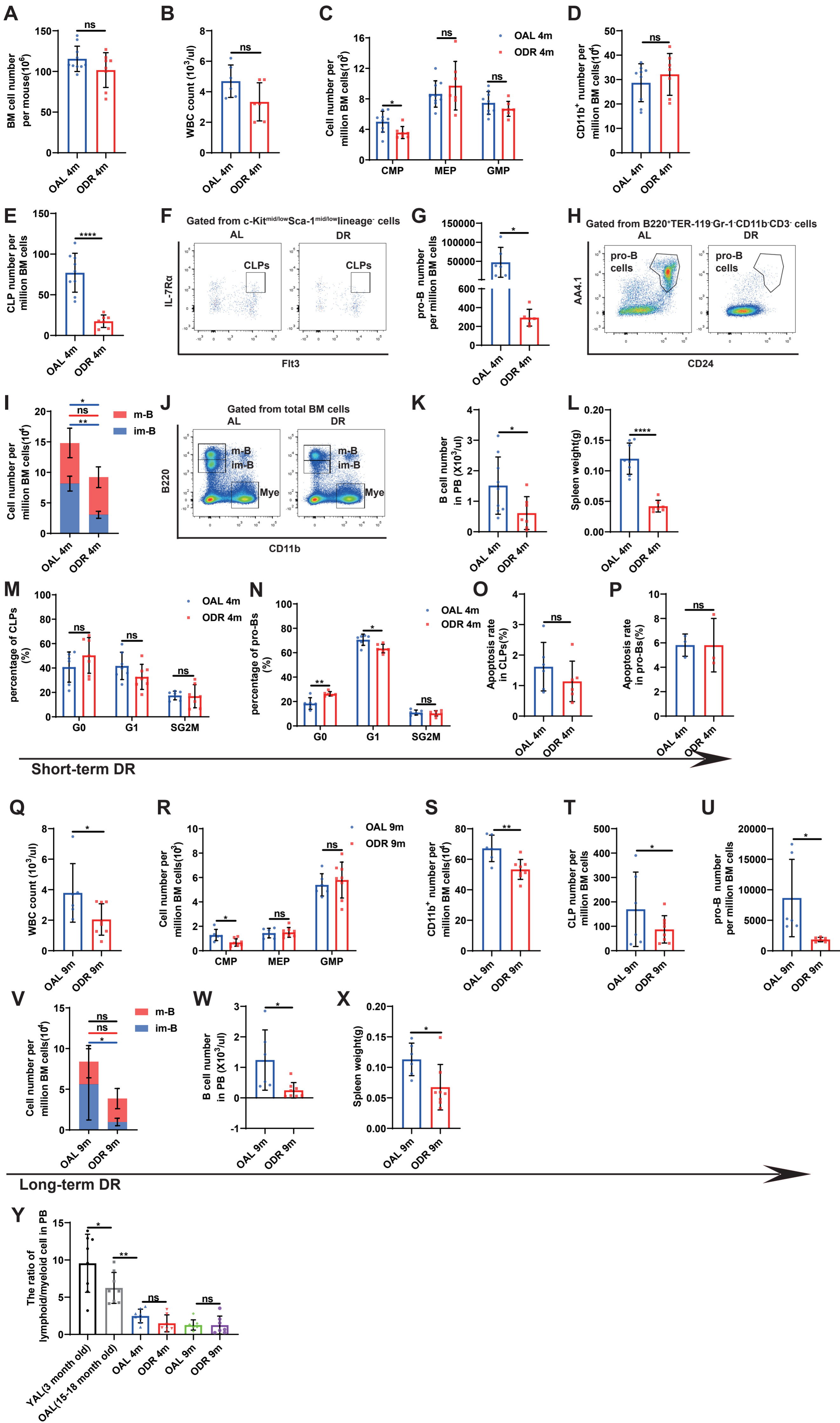

Tao and Wang et al\_Figure S2

Supplement: Supplementary file 2 [file ACEL-19-e13241-s002.pdf]

**A**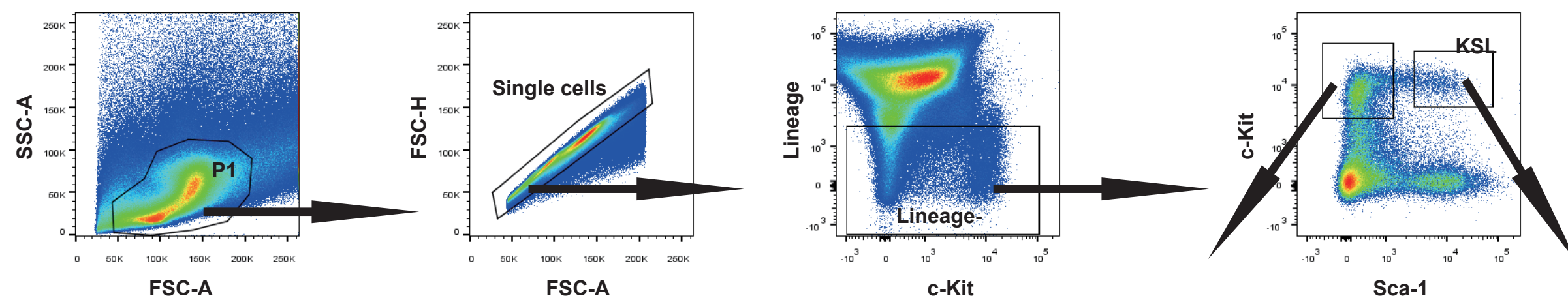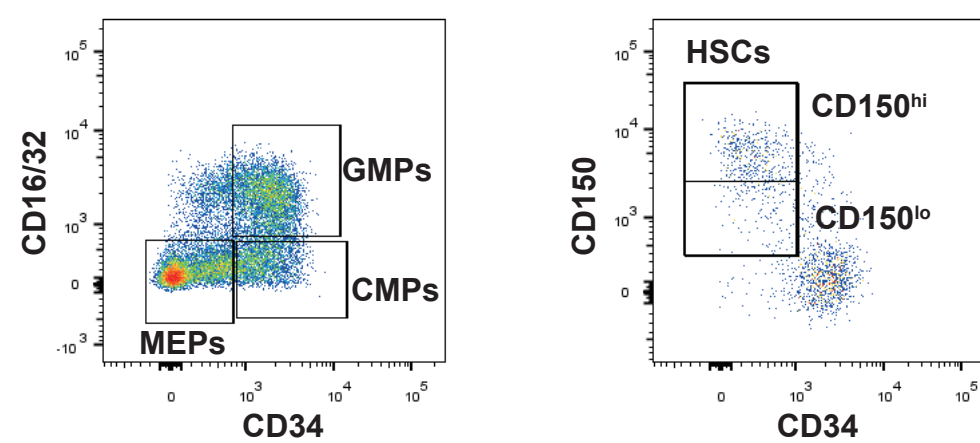**B**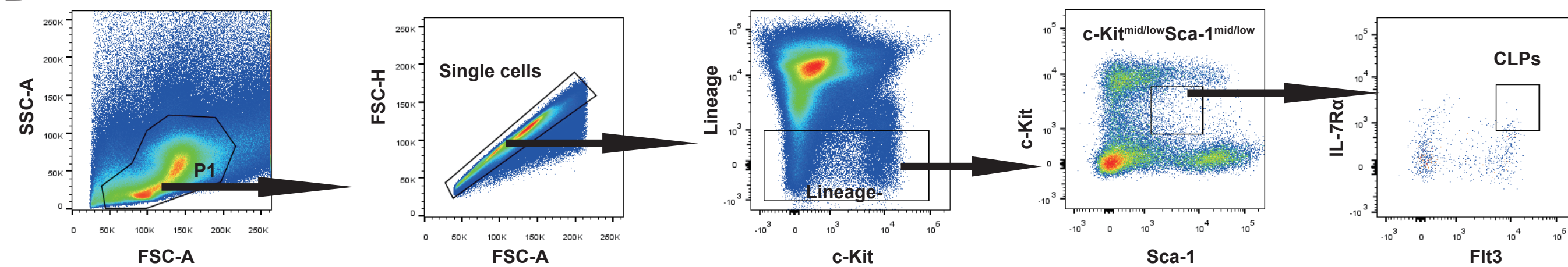**C**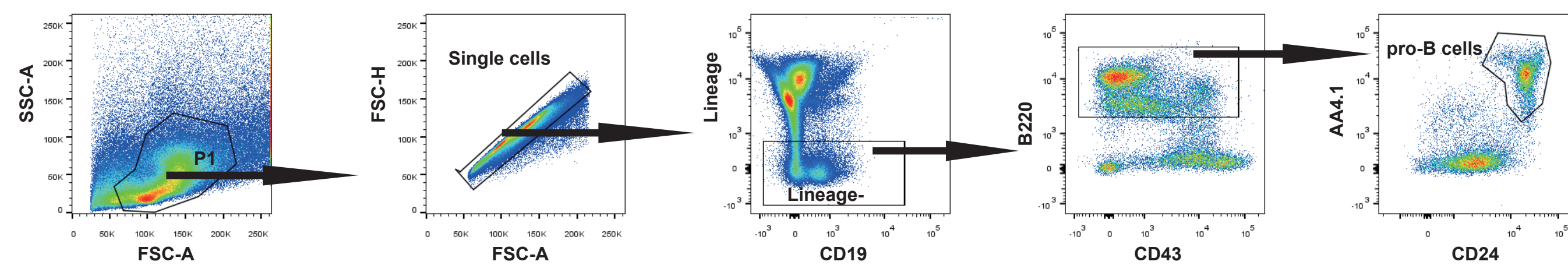**D**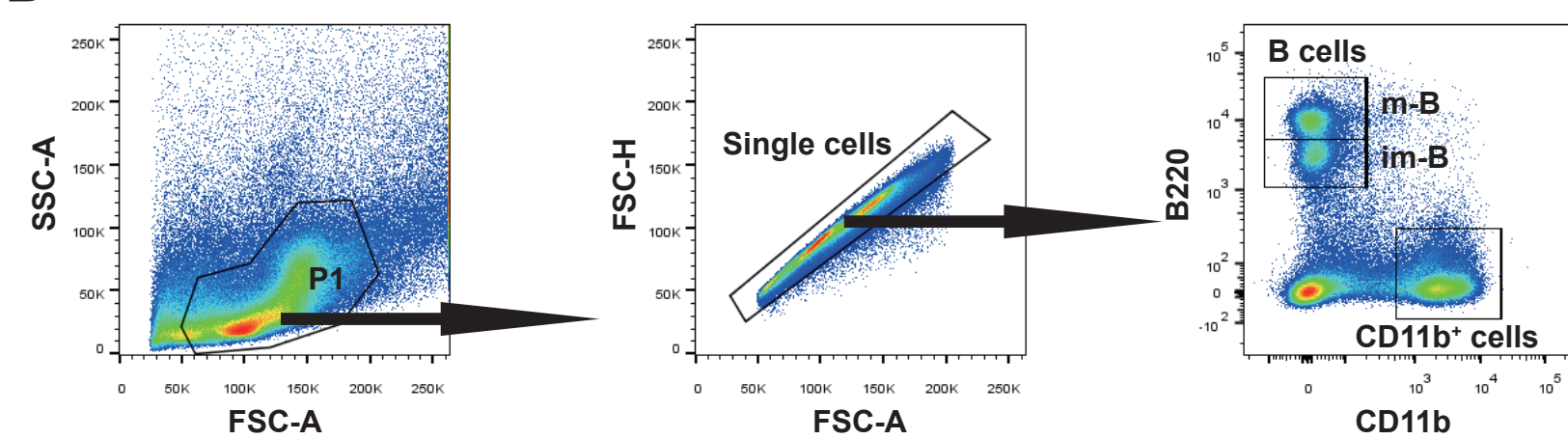**E**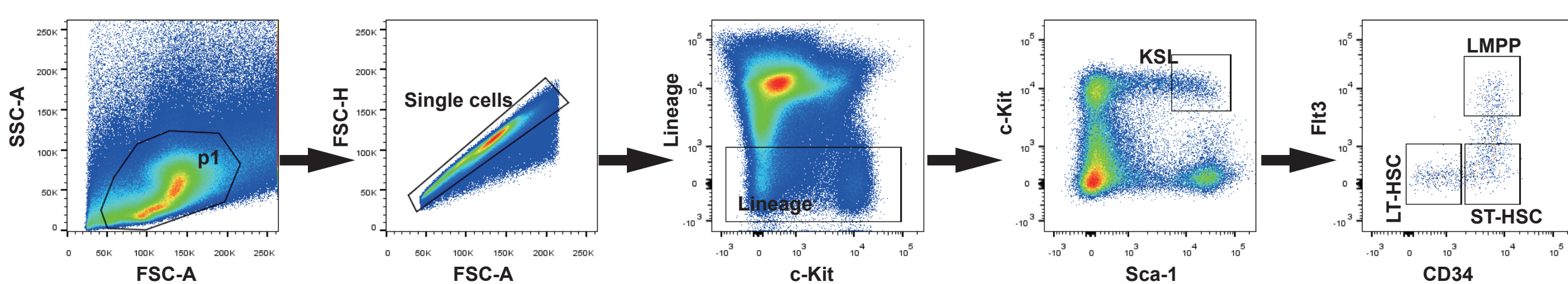**F**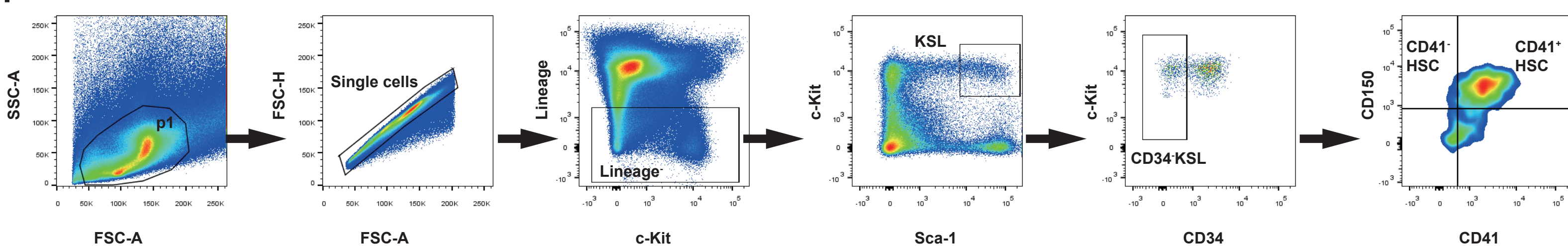

Supplement: Supplementary file 3 [file ACEL-19-e13241-s003.pdf]
