# Supplementary material for: Long‐term mid‐onset dietary restriction rejuvenates hematopoietic stem cells and improves regeneration capacity of total bone marrow from aged mice
Source: Aging Cell. 2020 Sep 15;19(10):e13241. doi: 10.1111/acel.13241 (PMC7576247; doi:10.1111/acel.13241)
Supplement: Supplementary file 4 [file ACEL-19-e13241-s004.docx]

**Table S1.Primer list of genes**

| **Gene** | **Forward Primers** | **Reverse Primers** |
| --- | --- | --- |
| β-actin | CTAAGGCCAACCGTGAAAAG | ACCAGAGGCATACAGGGACA |
| p21 | AACATC TCAGGGCCGAAA | TGCGCTTGGAGTGATAGAAA |
| C1pP | CTGCCCAATTCCAGAATCAT | TGTAGGCTCTGCTTGGTGTG |
| HSP10 | CCAAAGGTGGCATTATGCTT | TGACAGGCTCAATCTCTCCA |
| HSP60 | ACCTGTGACAACCCCTGAAG | TGACACCCTTTCTTCCAACC |
| SIRT7 | CCATGGGAAGTGTGATGATG | TCCTACTGTGGCTGCCTTCT |
| SIRT2 | TGGGCTGGATGAAAGAGAA | GGTCCACCTTGGAGAAGTCTG |
| NLRP3 | TCTTCCAGACTGGTGAACTGC | GTCCAGTTCAGTGAGGCTCC |
| Caspase 1 | AAGAAACATGCGCACACAGC | CCCTCAGGATCTTGTCAGCC |
