# Supplementary material for: Long‐term mid‐onset dietary restriction rejuvenates hematopoietic stem cells and improves regeneration capacity of total bone marrow from aged mice
Source: Aging Cell. 2020 Sep 15;19(10):e13241. doi: 10.1111/acel.13241 (PMC7576247; doi:10.1111/acel.13241)
Supplement: Supplementary file 5 [file ACEL-19-e13241-s005.docx]

**Supplemental figure 1**. **DR donor-derived bone marrow with same amount of HSCs improves hematopoietic regeneration after transplantation.** Mice were fed with DR or AL diet for 4 months. Number of bone marrow cells was adjusted to reach same amount of HSCs to be transplanted from AL and DR donor mice. Results show donor-derived chimerisms in primary (A) and secondary transplantations (B) (n=6-8). Results were displayed as mean ± SD. ****, P < 0.0001 by unpaired two-tailed Student’s t test.

**Supplemental figure 2**. **Mid-onset DR leads to suppression of B lymphopoiesis with minor effects on myelopoiesis.** (A-P) Mice were fed with DR or AL diet for 4 months. (A) Bone marrow cell counting (n=8-10). (B) Peripheral WBC counting (n=6). (C,D,E,G,I) Flow cytometry analysis of indicated populations (n=6-9). (F, H, J) Representative FACS plots of CLPs (F), pro-B cells (H) and B lymphocytes (J). (K) Quantification of peripheral B lymphocytes counted by absolute number of WBCs multiplied by frequencies of B lymphocytes determined by flow cytometry (n=7-9). (L) Weight of spleen (n=7-8). (M, N) Cell cycle status of CLPs (M) and pro-B cells (N) (n=6-7). (O, P) Percentage of apoptosis in CLPs (n=6).and pro-B cells (n = 3, from one experiment). (Q-X) Mice were fed with DR or AL diet for 9 months. (Q) Peripheral WBC counting (n=6-8). (R-V) Flow cytometry analysis of indicated populations (n=6-8). (W) Quantification of peripheral B lymphocytes counted by absolute number of WBCs multiplied by frequencies of B lymphocytes determined by flow cytometry (n=6-8). (X) Weight of spleen (n=6-8). (Y) The ratio of lymphoid versus myeloid cells (L/M) in peripheral blood (n=7-8). Results were displayed as mean ± SD. ns, not significant; *, P < 0.05; **, P < 0.01; ****, P < 0.0001. A-E,G,I,K-X:unpaired two-tailed Student’s t test; Y: one-way ANOVA. Data show results from 2 independent experiments unless otherwise indicated.

**Supplemental figure 3. Gating strategies of indicated populations.** (A) Gating strategy of HSCs and myeloid/erythroid progenitors in flow cytometry analysis. (B) Gating strategy of CLPs in flow cytometry analysis. (C) Gating strategy of pro-B cells in flow cytometry analysis. (D) Gating strategy of B cells, CD11b^+^ cells in flow cytometry analysis. (E) Gating strategy of LT-HSC, ST-HSC and LMPP in flow cytometry analysis. (F) Gating strategy of CD41^-^HSC and CD41^+^HSC in flow cytometry analysis.
